# Dual Roles of Extracellular Histone H3 in Host Defense: Its Differential Regions Responsible for Antimicrobial and Cytotoxic Properties and Their Modes of Action

**DOI:** 10.3390/antibiotics11091240

**Published:** 2022-09-13

**Authors:** Yuri Tanaka, Nanako Yamanaka, Izumi Koyano, Itaru Hasunuma, Tetsuya Kobayashi, Sakae Kikuyama, Shawichi Iwamuro

**Affiliations:** 1 Department of Biology, Faculty of Science, Toho University, Chiba 274-8510, Japan; 2 Division of Life Science, Graduate School of Science and Engineering, Saitama University, Saitama 338-8570, Japan; 3 Department of Biology, Faculty of Education and Integrated Arts and Sciences, Center for Advanced Biomedical Sciences, Waseda University, Tokyo 162-8480, Japan

**Keywords:** histone H3, antimicrobial activity, cytotoxicity, cell membrane destruction, ELEBA

## Abstract

Extracellular histones play a dual role—antimicrobial and cytotoxic—in host defense. In this study, we evaluated the antimicrobial and cytotoxic activities of histone H3 and identified the responsible molecular regions for these properties. Broth microdilution assays indicated that histone H3 exhibits growth inhibitory activity against not only Gram-negative and -positive bacteria but also fungi. Observations under scanning electron microscopy (SEM) revealed that histone H3 induced morphological abnormalities on the cell surface of a wide range of reference pathogens. MTT assays and SEM observations indicated that histone H3 has strong cytotoxic and cell lytic effects on mammalian normal, immortal, and tumor cell lines. Assays using synthetic peptides corresponding to fragments 1–34 (H3DP1), 35–68 (H3DP2), 69–102 (H3DP3), and 103–135 (H3DP4) of histone H3 molecule demonstrated that its antimicrobial activity and cytotoxicity are elicited by the H3DP2 and H3DP3 protein regions, respectively. Enzyme-linked endotoxin binding assays indicated that histones H3 and H3DP1, H3DP2, and H3DP4, but not H3DP3, exhibited high affinities toward lipopolysaccharide and lipoteichoic acid. Our findings are expected to contribute to the development of new histone H3-based peptide antibiotics that are not cytotoxic.

## 1. Introduction

Histones are major components of the nucleosome structure in eukaryotic cells and are involved in gene replication, transcriptional regulation, and DNA repair [1,2]. In addition to the nuclear histones, extracellular histones are also present in digestive fluids and circulating blood and are detected in connective tissue as well as outside the body [3]. These extracellular histones display cytotoxic effects on both prokaryotic and eukaryotic cells. Therefore, although they act as antimicrobial agents against pathogenic microorganisms, they also damage host cells [4].

The first report mentioning the antimicrobial activity of histones was published in 1942 [5]. Since then, histones and some fragmented histones have been found to have antimicrobial properties in arthropods (H2A, H2B, and H4) [6], fish (H1, H1-like, and fragmented H2A) [7,8,9,10], amphibians (H2B and fragmented H2A) [11,12], birds (H1, H2A, and H2B) [13,14], and mammalians, including humans (H1, H2A, H2B, and H4) [15,16,17,18]. At present, although only histone H3 has not yet been isolated from natural sources, all histone subtypes—H1, H2A, H2B, H3, and H4—have been shown to possess antibacterial and/or antifungal activities in vitro [19,20].

The five histone subtypes are functionally classified as linker (H1) and core (H2A, H2B, H3, and H4) histones and characteristically classified as lysine (Lys)-rich (H1, H2A, and H2B) and arginine (Arg)-rich (H3 and H4) histones. Interestingly, while Lys-rich histones penetrate the bacterial cell membrane and inhibit cell growth by binding to cytosolic nucleic acids, Arg-rich histones do not penetrate but remain on the cell surface; as a consequence, Arg-rich histones disrupt the cell membrane with bleb formation in a manner similar to general antimicrobial peptides (AMPs) [21,22]. Studies have demonstrated that histones do not necessarily need their full-length sequence, but only a partial sequence, to exert their antimicrobial activity. For example, the amino acid sequence of buforin I, an AMP isolated from the stomach extract of the Korean toad *Bufo bufo gargarizans*, is identical to the first 39 amino acids of histone H2A. The peptide is produced via the action of pepsin that is released into the gastric lumen [23]. Similarly, Lys-rich histone H2B and Arg-rich histones H3 and H4 inhibit *Escherichia coli* growth via digestion by its outer membrane proteinase T [12,21]. Furthermore, peptides synthesized according to the partial amino acid sequences of histones have been reported to exhibit antimicrobial activity [24,25,26]. Thus, histones can be used as templates for the design of novel AMPs.

In addition to their role as direct antimicrobial molecules, extracellular histones contribute to the host defense as components of neutrophil extracellular traps (NETs). These consist of nucleosomal histones and DNA released from neutrophils, which form extracellular fibers at the cell surface and bind to Gram-positive and -negative bacteria, resulting in the degradation of virulence factors and bacterial cell death [27]. NET formation induces neutrophil death; this process has been the focus of much attention as a new cell death process called NETosis, which is neither necrosis nor apoptosis [28]. Seventy percent of the proteins that form these NETs are core histones (H2A, H2B, H3, and H4), which are believed to contribute to biological defense through their antimicrobial properties as an NET component [29].

Sepsis is a systemic inflammatory response syndrome (SIRS) caused by pathogen infection. Extracellular histones are the major mediators of death during sepsis. Histones H3 and H4 have toxic effects on endothelial cells; the intravenous administration of histones to mice induces a pseudo-septic shock state with the accumulation of neutrophils in the lung, thrombus formation, and fibrotic changes; anti-histone H4 antibody ameliorates these symptoms [30]. A mixture of five calf histones (H1, H2A, H2B, H3, and H4) also exhibits toxic effects on endothelial cells; serum histone levels are higher in trauma and sepsis patients than in healthy donors, suggesting endothelial cell toxicity [31,32]. Extracellular histones increase cytokine levels and are mediators of death in sepsis through activation of nuclear factor-κB via toll-like receptors [33,34,35]. Thus, extracellular histones are metaphorically regarded as a double-edged sword in host defense: they contribute to the innate defense system with their direct antimicrobial activity and as NET components, but they also act as causative factors of disease. The current question is whether these two opposing effects originate from the same region within the histone molecules.

To our knowledge, no natural antimicrobial histone H3 or fragmented histone H3 have been identified yet. Histone H3 is referred to in many studies as a strong antibacterial and/or cytotoxic substance: however, few reports directly demonstrate these effects. Circulating histone H3 levels are associated with coagulopathy, multiple organ failure, and death in patients requiring intensive care because of infectious diseases [36]. In this study, we aimed to demonstrate the antimicrobial and cytotoxic activities of full-length histone H3 and to clarify which region of its protein sequence is responsible for each activity. Four synthetic peptides based on the amino acid sequence of human histone H3.1 (Supplementary Figure S1) were generated and subjected to antimicrobial, 3-(4,5-dimethylthiazol-2-yl)-2,5-diphenyltetrazolium bromide (MTT), morphological, and endotoxin-binding assays.

## 2. Experimental Results

### 2.1. Antimicrobial Activities of the Full-Length Histone H3 and SEM Analysis

**Figure 1:**
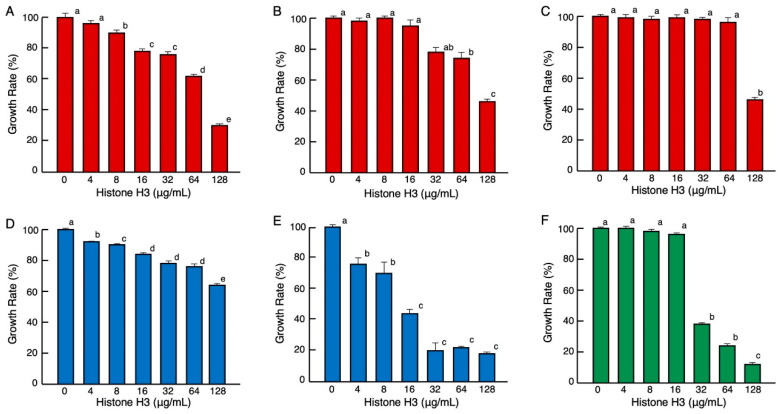
Effects of various concentrations of histone H3 on the growth of pathogenic Gram-negative bacteria (red columns) *E. aerogenes* (**A**), *S. enterica* (**B**), *P. aeruginosa* (**C**), Gram-positive bacteria (blue columns) *B. cereus* (**D***)*, *S. mutans* (**E**), and fungus (green columns) *C. albicans* (**F**). Cells of each microbial strain were incubated with two-fold serially diluted calf thymus histone H3 for 18 h (**A**–**D**), 36 h (**E**), or 24 h (**F**) at 37 °C. Each column and vertical line represent the mean and standard error of the mean, respectively (*n* = 4). The growth rates are expressed relative to the control (0 μg/mL histone H3). In all panels, values with the same letters (a–e) are not significantly different (*p* ≥ 0.05).

Antimicrobial assays showed that histone H3 was active against Gram-negative bacteria (*Enterobacter aerogenes*, *Salmonella enterica*, and *Pseudomonas aeruginosa*), Gram-positive bacteria (*Bacillus cereus* and *Streptococcus mutans*), and a fungal strain (*Candida albicans*). The minimum significant active concentrations were 8, 64, 128, 4, 4, and 32 μg/mL, corresponding to approximately 0.53, 2.1, 8.5, 0.26, 0.26, and 2.1 μM, respectively (Figure 1). Although the minimum significant active concentrations were different for each microorganism, these results demonstrated that histone H3 exhibited growth-inhibitory activities against a wide range of microbes, including aerobes (*P. aeruginosa, B. cereus*, and *C. albicans*) and facultative anaerobes (*E. aerogenes*, *S. enterica*, and *S. mutans*). The antimicrobial activity of histone H3 was stronger against the fungi than bacteria. To the best of our knowledge, this study is the first to demonstrate that histone H3 has growth-inhibitory effects on fungi.

Killing assays showed that 32 μg/mL of histone H3 reduced the cell viability of *E. aerogenes*, *S. enterica*, *P. aeruginosa*, *E. coli*, and *S. aureus* compared with controls (0 min) by 10% to 60% after 10 min of incubation, and by 0% to 20% after 30 min of incubation when (Supplementary Figure S3). These results suggest that histone H3 rapidly affected the bacterial cells. No significant effects on the number of *B. cereus* and *C. albicans* survivors were observed under these experimental conditions.

**Figure 2:**
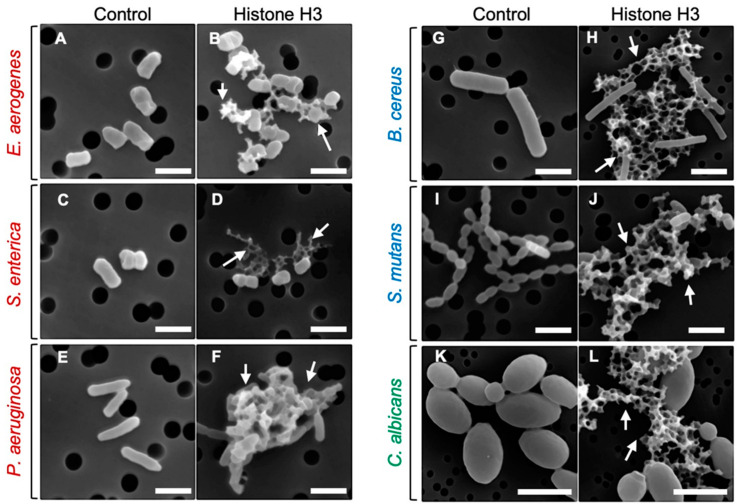
Scanning electron microscopy (SEM) images of bacterial and fungal cells following the treatment with histone H3. Aliquots of *E. aerogenes* (**A**,**B**), *S. enterica* (**C**,**D**), *P. aeruginosa* (**E**,**F**), *B. cereus* (**G**,**H**), *S. mutans* (**I**,**J**), or *C. albicans* (**K**,**L**) cultures at the mid-logarithmic growth phase were incubated with myoglobin (negative control) or histone H3 at 64 μg/mL for 1 h (**A**,**B**,**I**,**J**) or 4 h (**C**–**H**,**K**,**L**) at 25 °C, and were then examined using SEM. Abnormally shaped cells indicating cell surface destruction (arrows) are visible in each panel when compared with myoglobin-treated cells. Bars, 1 μm. Note that the black circles behind the cells are filter pores.

To investigate the mode of antimicrobial action of histone H3, we examined the morphological changes of microbial cells treated with histone H3 using a scanning electron microscope (SEM). As indicated in Figure 2, despite the different efficacy against different microbial strains, progression of morphological changes occurred on the surface of each microbial strain treated with 64 μg/mL of histone H3 compared with that of control cells (0 μg/mL). These changes were characterized by cell fragmentation. Histone H3 showed significant destructive effects on cell membranes and caused the cell lysis in all Gram-negative and -positive bacterial and fungal strains examined in this study. Under these experimental conditions, the cell membranes of Gram-positive bacteria were more severely damaged by histone H3 than those of Gram-negative bacteria. A longer treatment with histone H3 induced clump formation in these microbial cell strains. While many cells displayed remarkable morphological abnormalities, relative amounts of intact cells were also observed (Figure 2). Overall, histone H3 seemed to affect individual microbe cells.

### 2.2. Cytotoxic Activities of the Full-Length Histone H3 and SEM Analysis

**Figure 3:**
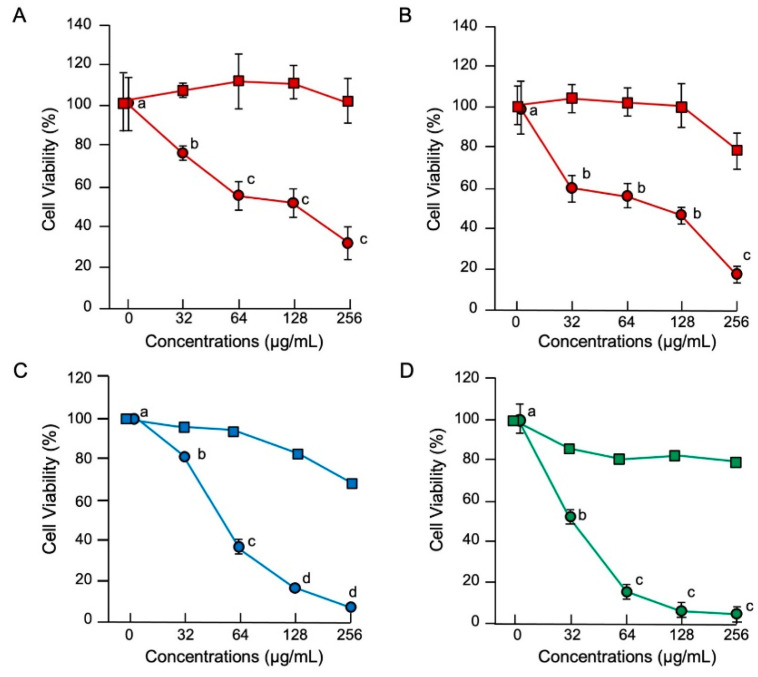
Cytotoxic effects of histone H3 in eukaryotic cells. Medium containing 1 × 10^4^ HUVEC (**A**) or HAEC (**B**) or 5 × 10^3^ COS7 (**C**) or HepG2 (**D**) cells in 500 μL aliquots were incubated with histone H3 (circles) or myoglobin (squares) at 0, 32, 64, 128, or 256 μg/mL for 24 h at 37 °C. Cell proliferation was determined using a standard MTT assay. Cell viability rates were calculated from MTT reduction values and are expressed relative to the control (0 μg/mL). Each point and vertical bar represent the mean and standard error of the mean, respectively (*n* = 4). In all panels, values with the same letters are not significantly different among histone H3 treatments (*p* ≥ 0.05). Cell lines of normal, immortal, and tumor cell lines are colored in red, blue, and green, respectively.

**Figure 4:**
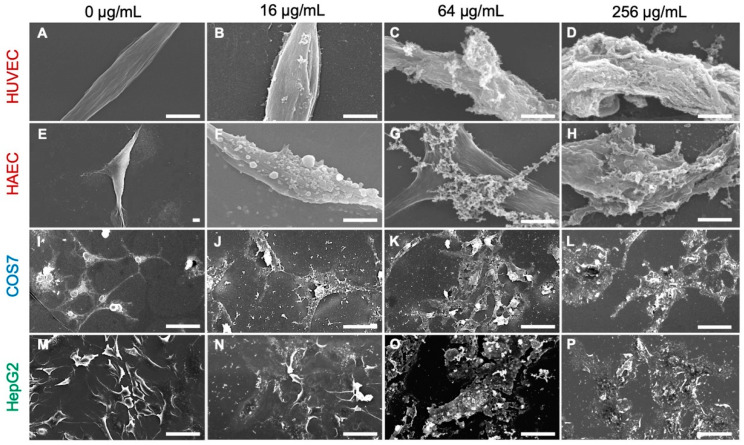
Scanning electron microscopy (SEM) images of mammalian cells following treatment with histone H3. Aliquots of HUVEC (**A**–**D**), HAEC (**E**–**H**), COS7 (**I**–**L**), or HepG2 (**M**–**P**) cells were incubated with histone H3 at 0, 32, 64, or 256 μg/mL for 24 h at 37 °C and then examined using SEM. Cell membrane destruction is visible in each panel of histone H3-treated cells when compared with control cells (0 μg/mL). Bars, 50 μm.

The MTT assay results demonstrated that histone H3 exhibited significant cytotoxic effects on normal cell lines of human umbilical vein endothelial (HUVEC), human aortic endothelial (HAEC) and calf pulmonary artery endothelium (CPAE) cells at a cell density of 1 × 10^4^ cells/well and on immortal (COS7) and tumor (HepG2) cell lines at 5 × 10^3^ cells/well (Figure 3). Observation of the cell morphology using SEM demonstrated that histone H3 exhibited strong cytotoxic effects on all the reference cell lines through cell membrane destruction in a dose-dependent manner (Figure 4). At a lower concentration (16 µg/mL), histone H3 induced the formation of bleb-like projections on the cell surface of both HUVEC and HAEC. At higher concentrations (≥64 µg/mL), further severe membrane destruction was observed. Overall, histone H3 showed stronger cytotoxic effects on normal cell lines than on tumor and immortal cell lines.

### 2.3. Identification of Antimicrobial and Cytotoxic Regions of Histone H3

**Figure 5:**
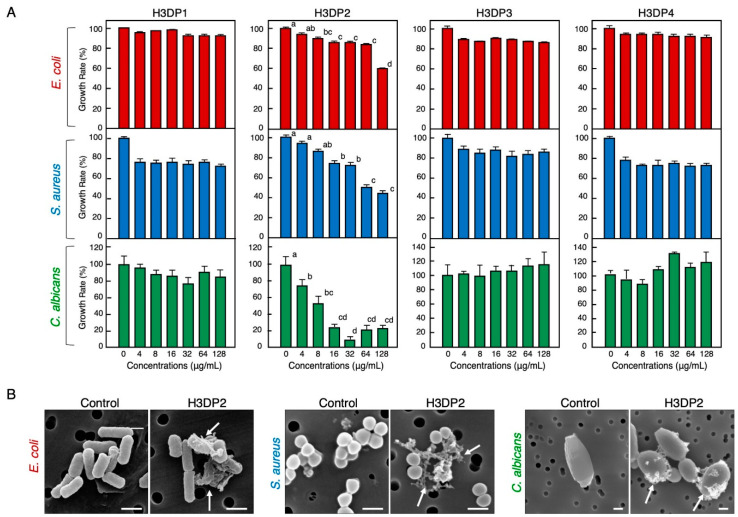
Effects of various concentrations of the four histone H3-derived peptides, H3DP1–4, on the growth of pathogenic bacteria *E. coli* and *S. aureus* and fungus *C. albicans* (**A**). Cells of each microbial strain were incubated with two-fold serially diluted H3DP1, H3DP2, H3DP3, or H3DP4 for 18 h (*E. coli*, *S. aureus*) or 24 h (*C. albicans*) at 37 °C. Each column and vertical bar represent the mean and standard error of the mean, respectively (*n* = 4). In all panels, values with the same letters are not significantly different (*p* ≥ 0.05). Scanning electron microscopy examination of bacteria and fungi following the treatment with H3DP2 (**B**). Aliquots of *E. coli*, *S. aureus*, or *C. albicans* cultures at the mid-logarithmic growth phase were incubated with H3DP2 or myoglobin (negative control) at 64 μg/mL for 1 h at 25 °C. Abnormally shaped cells similar to those observed in Figure 2 are visible in each panel (arrows). Bars, 1 μm.

To identify the antimicrobial region(s) of the histone H3 molecule responsible for its antimicrobial activity, we designed and purchased four histone H3-derived peptides (H3DPs), H3DP1–4, and subjected them to antimicrobial assays. As shown in Figure 5, only H3DP2 showed growth inhibitory activities against *E. coli*, *S. aureus*, and *C. albicans*, with minimum significant effective concentrations of 8, 16, and 4 µg/mL (corresponding to 1.9, 3.8, and 0.96 µM), respectively. As observed with full-length histone H3, H3DP2 treatment induced morphological abnormalities in *E. coli*, *S. aureus*, and *C. albicans*. 

**Figure 6:**
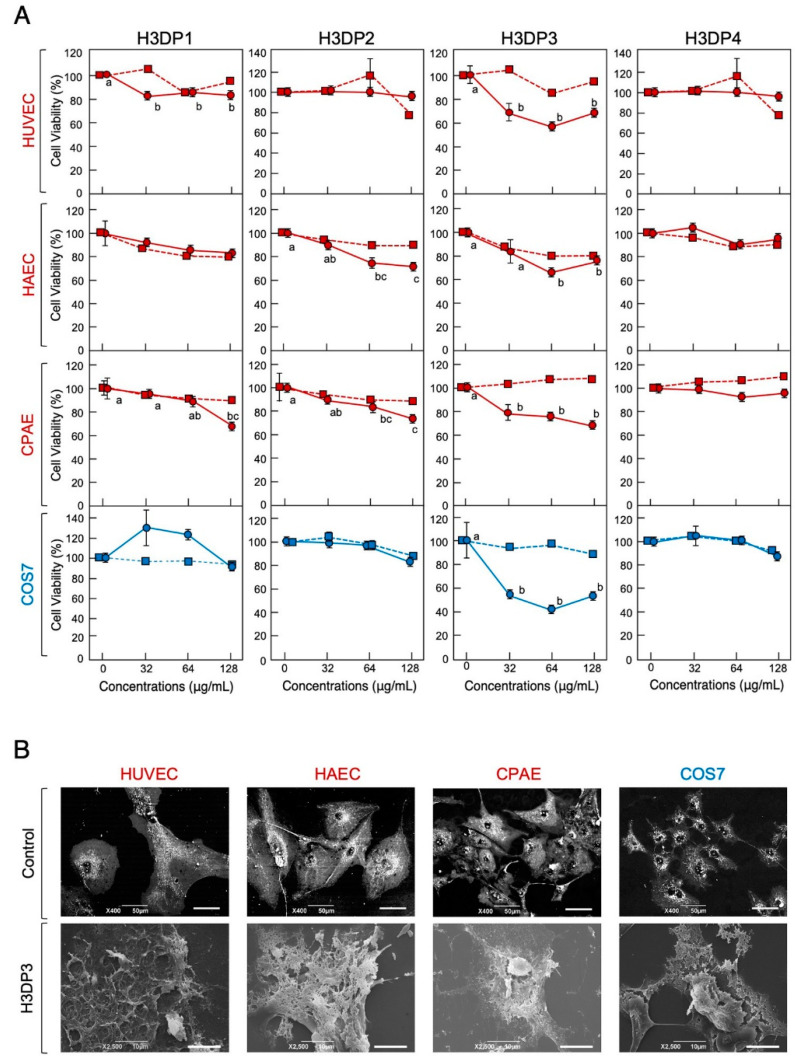
Cytotoxic effects of H3DP1, H3DP2, H3DP3, or H3DP4 on eukaryotic cells (**A**). Medium containing 1 × 10^4^ HUVEC, HAEC, or CPAE or 5 × 10^3^ COS7 cells in 500 μL aliquots were incubated with each peptide (circles) or myoglobin (squares) at 0, 32, 64, or 128 μg/mL for 24 h at 37 °C. Cell proliferation was determined using a standard MTT assay. Cell viability rates were calculated from MTT reduction values and are expressed relative to the control (0 μg/mL). Each point and vertical bar represent the mean and standard error of the mean, respectively (*n* = 4). In each panel, values with the same letters are not significantly different (*p* ≥ 0.05). Scanning electron microscopy (SEM) examination of mammalian cells following treatment with H3DP3 (**B**). Aliquots of HUVEC, HAEC, CPAE, or COS7 cells were incubated with H3DP3 or myoglobin (negative control) at 64 μg/mL for 24 h at 37 °C and were then examined using SEM. Bars, 50 μm (control) or 10 μm (H3DP3).

Next, we performed an MTT assay using the four H3DPs to identify the region(s) of histone H3 responsible for its cytotoxic properties. H3DP3 displayed cytotoxic effects on the normal cell lines HUVEC, HAEC, and CPA and immortal cell line COS7 at cell densities of 1 × 10^4^ and 5 × 10^3^ cells/well, respectively; peptide concentrations higher than 32 μg/mL (8.3 μM) for HUVEC, CPA, and COS7 and 64 μg/mL (16.6 μM) for HAEC induced significant toxicity (Figure 6). H3DP3 exhibited cell membrane-destructive activity and caused morphological abnormalities in all reference cell lines. H3DP1 and H3DP2, but not H3DP4, displayed slight but statistically significant toxicity to normal cell lines at higher concentrations. Overall, we conclude that the antimicrobial and cytotoxic activities of histone H3 originate from different regions, mainly those corresponding to H3DP2 and H3DP3, respectively. 

### 2.4. Bacterial Endotoxin Binding Ability of Histone H3 and Its Related Peptides

**Figure 7:**
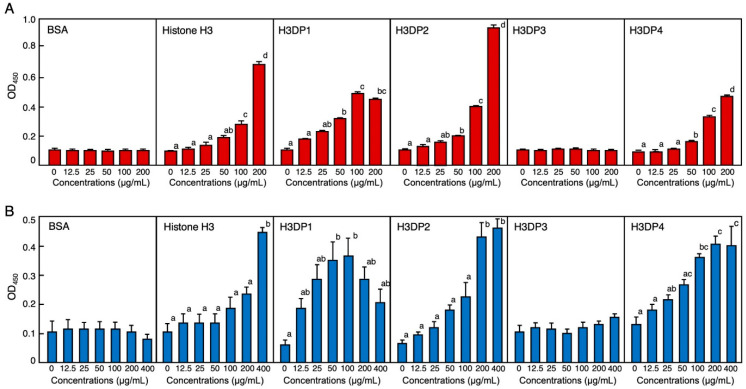
Evaluation of binding abilities of bovine serum albumin (BSA), histone H3, H3DP1, H3DP2, H3DP3, and H3DP4 to the bacterial endotoxins lipopolysaccharide (LPS) ((**A**); red columns) and lipoteichoic acid (LTA) ((**B**); blue columns) using enzyme-linked endotoxin binding assay (ELEBA). Serially diluted proteins or peptides were individually coated onto 96-well microplates and incubated with biotin-LPS or biotin-LTA for 4 h at 25 °C. The peptides-bound biotin-LPS or biotin-LTA were detected by incubation with HRP-labeled streptavidin followed by incubation with the ELISA POD substrate in the TMB solution. The reaction products were measured at 450 nm. Each column and vertical bar represent the mean and standard error of the mean, respectively (*n* = 4). In each panel, values with the same letters are not significantly different (*p* ≥ 0.05).

The results from the enzyme-linked endotoxin binding assay (ELEBA) revealed that histone H3 is capable of binding to both lipopolysaccharide (LPS) and lipoteichoic acid (LTA), the Gram-negative and -positive bacterial surface molecules, respectively. This observation suggests that histone H3 may capture bacterial cells by binding to these molecules (Figure 7). When considering the diluted concentrations of biotinylated LPS (biotin-LPS) and biotinylated LTA (biotin-LTA) into consideration, ELEBA results indicated that histone H3 had a higher affinity for LTA than for LPS. We previously detected the LTA-binding ability of histone H3 at a concentration of 200 μg/mL using a mobility shift assay [22]. In this study, we observed the LTA-binding ability of histone H3 at a concentration of 400 ng/mL using ELEBA. The assay results also revealed that H3DP2 had a higher affinity for LPS and LTA in a dose-dependent manner. In addition, H3DP1 and H3DP4 were also shown to possess an affinity to LPS and LTA. Neither growth-inhibitory activity against the microbes nor endotoxin-binding activity were detected for the acidic peptide H3DP3 (Figure S1). This may be one of the reasons for the lack of antimicrobial activities of H3DP3.

## 3. Discussion

Even though some studies indicate that histone H3 exhibits antimicrobial activity against a broad spectrum of pathogenic microorganisms, this antimicrobial spectrum is still unclear. To date, only the action of commercial calf histone H3 against *E. coli* and *S. aureus* has been reported [21,22]. In this study, we initially aimed to obtain basic data on the antimicrobial activity of full-length histone H3. Although the effective concentrations varied among bacterial strains, histone H3 clearly showed significant antimicrobial activity against three Gram-negative (*E. aerogenes*, *S. enterica*, and *P. aeruginosa*) and two Gram-positive (*B. cereus* and *S. mutans*) bacterial strains as well as one fungus (*C. albicans*). All antimicrobial assays were conducted under identical experimental conditions (e.g., temperature and culture broth). However, standard assay conditions are not necessarily suitable for all bacteria [37]: each bacterial cell has a different doubling time, with some growing faster and others slower; some are bifid in their proliferation, while others are endospore-forming [38]. Therefore, analysis of antimicrobial activity using SEM can give important information on a potential membrane-disrupting action. SEM revealed that histone H3 acts on the plasma membrane of pathogenic microorganisms, including fungi, and causes morphological abnormalities and disruption of the cell integrity. 

Although there are no obvious consensus amino acid sequences among AMPs, most are hydrophobic and cationic and have the propensity to form an amphipathic helical conformation in a membrane-mimetic environment [39,40,41]. Each of these features affects the interaction between AMPs and bacterial membranes. Each histone has regions within its protein structure that are responsible for its antimicrobial activity. As mentioned in the Introduction section, there are many examples of peptide fragments resulting from the fragmentation of histones (except for histone H3) that exhibit antimicrobial activity. For histone H3, no peptide fragment with antimicrobial activity has been found directly from a biological source; however, because it can be digested by *E. coli* and *S. aureus* cell lysates [12,22], it is possible that the resulting fragment containing H3DP2-related sequences is responsible for the antimicrobial activity. Therefore, selecting regions with this AMP-like sequence may allow the identification of template sequences for creating new AMPs. Secondary structure and helical wheel analysis (Figure S1) suggested that among H3DP1–4, H3DP2 fulfills these characteristics best, but not H3DP3, which is the only anionic peptide. These features are reflected in their antimicrobial properties. Previous reports have shown that a synthetic peptide generated based on the *Xenopus* 39–58 histone H3 sequence (HRYRPGTVALREIRRYQKST) containing an amino acid sequence corresponding to our active peptide H3DP2 displayed antimicrobial activity against Gram-negative (*E. coli* and *Serratia marcescens*) and Gram-positive (*Enterococcus faecalis*, *S. aureus*, and *Bacillus subtilis*) bacteria, although this was not validated for fungi [24,25]. Despite the fact that the activity of H3DP2 is weaker than that of histone H3, its mode of action and antimicrobial spectrum are preserved in the peptide, suggesting that H3DP2 is the region responsible for the antimicrobial properties of histone H3.

As mentioned above, antimicrobial activity has also been reported for some peptides synthesized by selecting partial sequences of each of the other histones. However, these peptides corresponded only to a selected part of the total sequence and the other regions were not used in the assays. In addition to the inhibition of microbial growth, which is generally used as an indicator, other mechanisms of antimicrobial activity exist, such as bacterial aggregation or neutralization via binding to bacterial toxins. To investigate the broad antimicrobial properties of histone H3, the endotoxin-binding ability of full-length histone H3 and H3DPs was analyzed using ELEBA. Cationic histone H3 showed the strongest affinity for LPS and LTA, while cationic peptides H3DP1, H3DP2, and H3DP4 showed strong or moderate affinity, suggesting that the corresponding regions of H3DP1 and H3DP4 in histone H3 may contribute to capturing the pathogenic microbes via binding to their cell surface substances. The anionic peptide H3DP3 was not capable of binding to these endotoxins, suggesting that this region may not be directly involved in the antimicrobial action of histone H3.

The cytotoxic properties of histones have been actively studied, particularly in vivo, since their association with sepsis has been reported. Free histone levels are increased in the serum of patients with severe trauma, pancreatitis, and sepsis, and the cytotoxic effects of these circulating histones induce organ damage and death [20]. In in vitro microscopic studies, cultured vascular endothelial cells and leukocytes treated with 25–50 μg/mL histone H3 for 6 h shrunk and died [42]. These active concentrations agree with the MTT assay that resulted from this study. In this assay, MTT reduction, expressed by NAD(P)H-dependent cellular oxidoreductase enzyme activity, reflects the number of viable cells; therefore, rapidly dividing cells, such as tumor and eternal cell lines, exhibit higher rates of MTT reduction than cells with a low metabolism, such as a normal cell line. When we first used the same cell density (1 × 10^4^) that was initially used for all the reference cell lines, cytotoxicity could not be detected for histone H3 on HepG2 and COS7 cells (data not shown). However, when the cell density was reduced to half (5 × 10^3^), histone H3 concentration-dependent cytotoxicity against these cell lines was observed. Under identical cell density conditions, we performed an MTT assay for H3DP1–4 on these cell lines and detected obvious cytotoxic activity for H3DP3 via cell membrane destruction, similarly to the full-length histone H3. According to these results, the cytotoxic activity of H3DP3 was relatively weaker than that of histone H3; however, SEM observations revealed severe cell destruction by H3DP3. The results of a series of experiments indicate that the molecular regions of antimicrobial activity and the regions of cytotoxicity are distinct in histone H3.

The relationship between the cytotoxic effects of histones and disease has drawn worldwide attention and is being actively investigated, with NETs being implicated in the pathogenesis of inflammatory diseases. Activated protein C, a major regulatory factor of blood coagulation, cleaves histones and is protective in models of sepsis and after severe trauma. Histones present in NETs are protected against degradation by activated protein C [43]. Marsman et al. demonstrated that factor VII–activating protease cleaves histones and protects against histone-induced cytotoxicity both in vitro and in vivo [44]. The lower toxicity of H3DP3 compared with that of full-length histone H3 may be related to the fact that this proteolytic degradation of histone H3 reduced its cytotoxicity. Masking this region with antibodies or histone-binding substances, such as DNA and heparin [45], may also reduce the inflammatory response caused by circulating histone H3. 

## 4. Materials and Methods

All experiments were approved by the Toho University Biosafety Committee for Pathogens and were performed by authorized investigators.

### 4.1. Synthetic Peptides

The four synthetic peptides H3DP1, H3DP2, H3DP3, and H3DP4, corresponding to sequence fragments 1–34 (ARTKQTARKSTGGKAPRKQLATKAARKSAPATGG), 35–68 (VKKPHRYRPGTVALREIRRYQKSTELLIRKLPFQ), 69–102 (RLVREIAQDFKTDLRFQSSAVMALQEACEAYLVG), and 103–135 (LFEDTNLCAIHAKRVTIMPKDIQLARRIRGERA), respectively, of human histone H3.1 (NCBI accession No. NP_003520.1), respectively, were purchased from GL Biochem (Shanghai, China) with ≥90% purity (Supplementary Figure S2). Amino acid sequences and compositions, molecular weights, deduced isoelectric points, and net positive charge values of the peptides were analyzed using Genetyx-Mac version 15.0.1 software (Software Development Corporation, Osaka, Japan). The secondary structures and helical wheel projections of the peptides were predicted using the online tools PSIPRED (http://bioinf.cs.ucl.ac.uk/psipred/, accessed on 30 March 2022) and NetWheels (http://lbqp.unb.br/NetWheels/, accessed on 30 March 2022), respectively. The structural and biochemical characteristics of the peptides are shown in Figure S1.

### 4.2. Bacterial and Fungal Cell Strains

The pathogenic Gram-negative bacterial strains *E. coli* (JCM5491), *E. aerogenes* (JCM1235), *S.* *enterica* (JCM1652), and *P. aeruginosa* (JCM6119), Gram-positive bacterial strains *S. aureus* (JCM2874), *B. cereus* (JCM2152), and *S. mutans* (JCM5705), and the fungal *C. albicans* (JCM2085) strain were purchased from the Japan Collection of Microorganisms (Riken Bioresource Center, Tsukuba, Japan). Cells were grown on appropriate agar plates and then inoculated into the growth medium recommended by the manufacturer for secondary culture for antimicrobial assays. All experiments were approved by the Toho University Biosafety Committee for Pathogens and were performed by authorized investigators. 

### 4.3. Mammalian Cell Lines

HUVEC and HAEC (normal cell lines) were purchased from Toyobo (Osaka, Japan). African green monkey kidney-derived COS7 cells (an immortal cell line), human liver hepatocellular carcinoma-derived HepG2 cells (a tumor cell line), and CPAE cells (a normal cell line) were purchased from the Health Science Research Resource Bank (Osaka, Japan). HUVEC and HAEC were cultured in EGM-2 BulletKit (Lonza, Walkersbille, MD, USA). COS7 and HepG2 cells and CPAE cells were cultured in Dulbecco’s modified Eagle medium (Nissui, Tokyo) and minimum essential medium, respectively, supplemented with 10% fetal bovine serum (FBS; Sigma-Aldrich, St. Louis, MO, USA) and antibiotics (100 U/mL penicillin and 100 μg/mL streptomycin; Life Technologies, Carlsbad, CA, USA). All cell strains were cultured at 37 °C under 5% CO_2_–95% air conditions.

### 4.4. Antimicrobial Assay 

The antimicrobial activity of histone H3 and H3DPs was determined using a broth microdilution assay according as described in our previous study [45]. Briefly, two-fold serially diluted calf thymus histone H3 (Sigma-Aldrich) or each peptide were incubated determined in Mueller–Hinton broth (100 μL) with log-phase cultures (5 × 10^3^ colony forming units/well at the final concentration) of *E. aerogenes*, *S. enterica*, *P. aeruginosa*, *B. cereus*, *S. mutans*, or *C. albicans* in 1% BSA-coated 96-well microtiter cell culture plates at 37 °C in the air with 150 rpm shaking. The final concentrations of histone H3 or the peptides ranged from 0 to 128 μg/mL. After incubation for 18 h for *E. aerogenes*, *S. enterica*, *P. aeruginosa*, and *B. cereus*, for 36 h for *S. mutans*, and 24 h for *C. albicans*, the absorbance of each well was measured at 595 nm using an iMark microtiter plate reader (Bio-Rad, Hercules, CA, USA). The antimicrobial assays were performed using ampicillin (100 μg/mL) as a positive control, which reliably inhibited the growth of all microorganisms tested (data not shown). For the killing assay, log-phase cultures were diluted and adjusted to 0.5 × McFarland standard with phosphate-buffered saline (PBS), and again diluted to 1:100. Each 10 µL aliquot of the microbe suspensions was incubated with 10 µL of histone H3 (64 µg/mL) at 37 °C for 10 and 30 min with 150 rpm shaking, then serially diluted, plated on tryptic soy agar plates, and incubated at 37 °C overnight; bacterial colonies were then counted. Three replicates were performed and the average values calculated. 

### 4.5. Cytotoxic Assay

To assess the cytotoxic effects of histone H3 and H3DPs on eukaryotic cells, a standard MTT assay was performed as described in our previous study [46]. Briefly, as references, 1 × 10^4^ HUVEC, HAEC, or CPAE cells and 5 × 10^3^ COS7 or HepG2 cells were cultured per well on a collagen-coated 96-well microtiter cell culture plate (Thermo Fisher Scientific, Waltham, MA, USA) containing 100 μL of the appropriate medium supplemented with 10% FBS and antibiotics at 37 °C overnight in an atmosphere of 5% CO_2_. The medium was then replaced with a fresh medium containing histone H3 or each H3DP at a final concentration of 0, 32, 64, and 256 μg/mL (histone H3 and lysozyme) or 0, 32, 64, and 128 μg/mL (H3DP1–4) and further incubated for 24 h. After incubation, the medium was replaced with 100 μL of fresh medium containing 0.5% MTT (Wako, Osaka, Japan) and incubated for 4 h in the dark, then aliquots (100 μL) of a lysis buffer, 6 N HCl/isopropanol (0.34/99.66, *v*/*v*) were added and incubated overnight under identical conditions. Finally, the absorbance of the specimens at 570 nm was measured using a microtiter plate reader.

### 4.6. Scanning Electron Microscopy

For antimicrobial experiments, the bacterial and fungal cells were grown in 1.5 mL tubes with 500 μL of Luria Bertani broth to an optical density of 0.6 at 600 nm, and then incubated with histone H3, H3DP2, or myoglobin (negative control) [21] at a concentration of 64 μg/mL for 1 and 4 h at 25 °C. After incubation, the cells were harvested by gentle centrifugation, prefixed with 2.5% glutaraldehyde for 1 h, fixed in 1% OsO_4_ for 1 h, and washed with phosphate buffer. The cells were dehydrated in an ethanol series (50%, 70%, 90%, 95% and 100%) on a nano-percolator filter (JEOL, Tokyo, Japan) followed by incubation with ethanol/t-butyl alcohol (1:1) for 1 h and three rounds of incubation with 100% t-butyl alcohol for 20 min. The samples were frozen at 4 °C, lyophilized, coated with gold (15 nm) using Quick Coater SC701 (Sanyu Electron, Tokyo, Japan), and then observed using a JSM-6390LV scanning electron microscope (JEOL). For cytotoxic experiments, HUVEC, HAEC, COS7, and HepG2 cells were pre-cultured on poly-L-lysine-coated 12-mm coverslips (Corning) placed in each well of a 24-well cell culture plate followed by incubation with histone H3, H3DP3, or myoglobin under the same experimental conditions described in the cytotoxic assay section. After the incubation, the SEM samples were prepared as described above and subjected to SEM analysis. Additional details are described in our previous report [46].

### 4.7. Enzyme-Linked Endotoxin Binding Assay

To assess the binding ability of histone H3 and H3DPs to bacterial endotoxins, such as LPS and LTA, we performed an ELEBA as described in our previous study [46]. Briefly, each well of a 96-well Nunc Immobilizer Amino microtiter plate (Thermo Fisher Scientific) was filled with 100 μL of serially-diluted histone H3 or each H3DP (0–2 mg/mL in 100 mM carbonate buffer, pH 9.6), and incubated overnight at 4 °C with gentle agitation for coupling, followed by post coupling with 10 mM ethanolamine in carbonate buffer for 1 h at 4 °C. The wells were blocked with Blocking reagent for ELISA (Sigma-Aldrich) and incubated for 1 h at 25 °C. The wells were aspirated, washed with PBS (pH7.2) containing 0.05% (*w*/*v*) Tween 20 (PBS-T), and incubated with biotin-LPS (InvivoGen, San Diego, CA, USA) from *E. coli* O111:B4 or *S. aureus* biotin-LTA. After incubation with biotin-LPS or biotin-LTA, the wells were aspirated and washed with PBS-T. Then, 100 μL of streptavidin-conjugated horseradish peroxidase (SA-HRP; PerkinElmer, Waltham, MA, USA) diluted in the Blocking reagent was added, followed by incubation for 30 min at 25 °C. The wells were aspirated, washed with PBS-T and PBS, and then reacted with 100 μL of the substrate solution in ELISA POD Substrate TMB kit (Nakalai Tesque, Kyoto, Japan) for 15 min at 25 °C in the dark. The reaction was stopped by the addition of 100 μL of 1 M H_2_SO_4_. Finally, the absorbance of the specimens was measured at 450 nm using a microtiter plate reader. 

### 4.8. Statistical Analyses

Statistical analyses for the antimicrobial and cytotoxic assays were performed using analysis of variance (ANOVA) followed by multiple comparisons using Scheffé’s *F* test. For the killing assay, a student’s *t*-test was performed. A value of *p* < 0.05 was considered to be statistically significant. 

## 5. Conclusions

The present study demonstrates that histone H3 exhibits a broad antimicrobial spectrum, including Gram-negative and -positive bacteria and fungi. The mode of antimicrobial action is thought to occur via binding to surface molecules of pathogens and subsequent disruption of cell membranes; H3DP2 contains the amino acid sequence responsible for its antimicrobial activity. Histone H3 also exhibits strong cytotoxicity against normal, immortal, and tumor cell lines via cell membrane destruction; H3DP3 contains the amino acid sequence responsible for this cytotoxic activity. These findings contribute to the development of new histone H3-based AMPs that are devoid of cytotoxicity and to the development of therapies for SIRS caused by infectious diseases and circulating histone H3 that maintains its antibacterial properties of histone H3.

## Data Availability

Not applicable.

## Supplementary Materials

The following supporting information can be downloaded at: https://www.mdpi.com/article/10.3390/antibiotics11091240/s1, Figure S1: Killing kinetics of histone H3 against bacterial cell and fungal strains. Figure S2: Purity certification by HPLC and mass spectrometry values for the synthetic peptides, H3DP1 (A), H3DP2 (B), H3DP3 (C), and H3DP4 (D). Figure S3: Amino acid sequences and compositions, molecular weight, isoelectric points, net charges, and predicted secondary structures and helical wheel projections of the histone H3-derived peptides, H3DP1–4.
