# Supplementary material for: Dual Roles of Extracellular Histone H3 in Host Defense: Its Differential Regions Responsible for Antimicrobial and Cytotoxic Properties and Their Modes of Action
Source: Antibiotics (Basel). 2022 Sep 13;11(9):1240. doi: 10.3390/antibiotics11091240 (PMC9495139; doi:10.3390/antibiotics11091240)
Supplement: Supplementary file 1 [file antibiotics-11-01240-s001.zip › YT.Figure.S2.Prf.pdf]

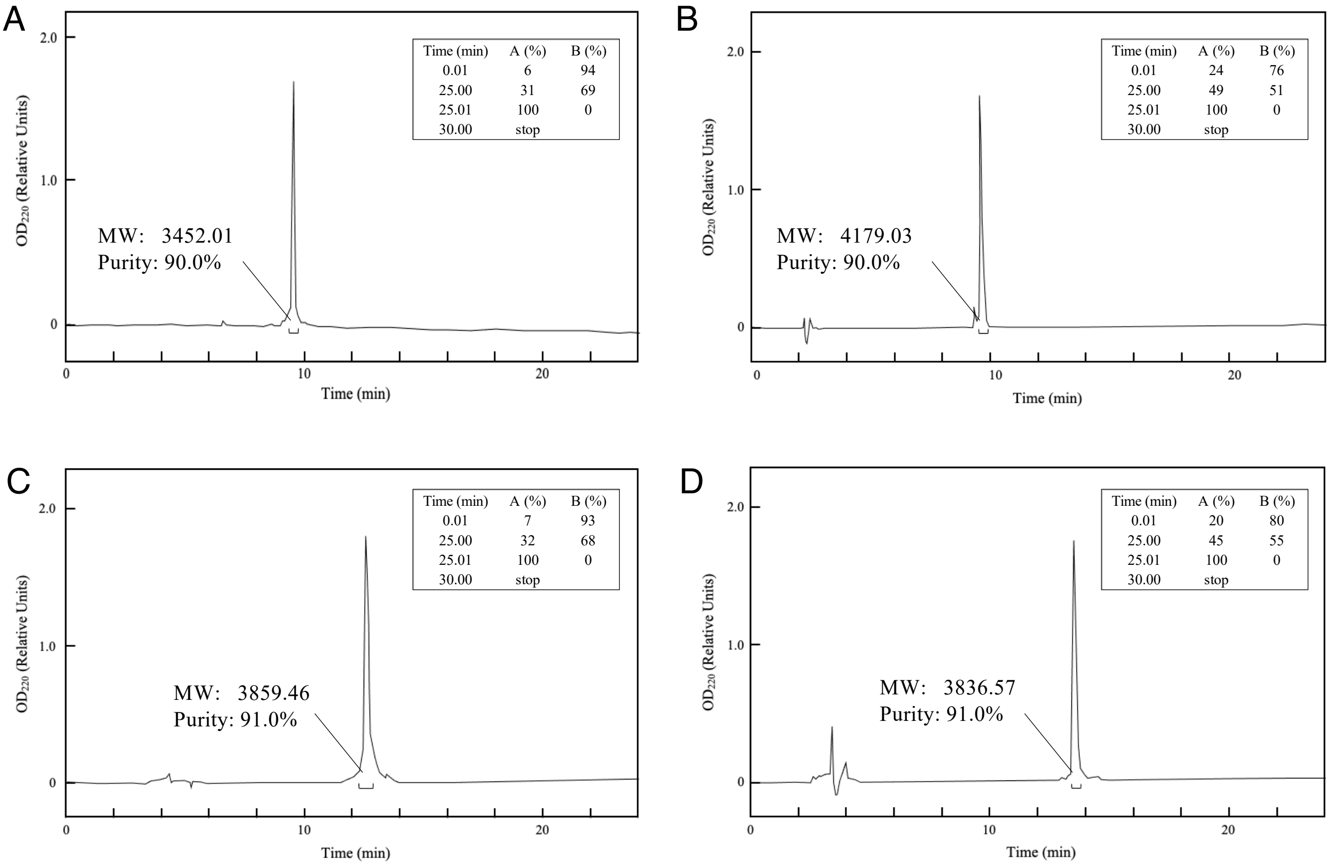

Figure S2. Purity certification by high performance liquid chromatography and mass spectrometry values for the synthetic peptides, H3DP1 (A), H3DP2 (B), H3DP3 (C), and H3DP4 (D). The peptides were chromatographed on an (4.6 × 250 mm) Inertsil ODS-SP column (GL Science, Tokyo) with a 1.0 mL/min flow rate. The gradient conditions of solvents A (0.1% trifluoroacetic acid in 100% acetonitrile) and B (0.1% trifluoroacetic acid in 100% water) are shown in the squares in each panel. Calculated molecular weight (MW) was determined by the supplier (GL Biochem) using mass spectrometry.
