# Supplementary material for: Dual Roles of Extracellular Histone H3 in Host Defense: Its Differential Regions Responsible for Antimicrobial and Cytotoxic Properties and Their Modes of Action
Source: Antibiotics (Basel). 2022 Sep 13;11(9):1240. doi: 10.3390/antibiotics11091240 (PMC9495139; doi:10.3390/antibiotics11091240)
Supplement: Supplementary file 1 [file antibiotics-11-01240-s001.zip › YT.Figure.S3.Prf.pdf]

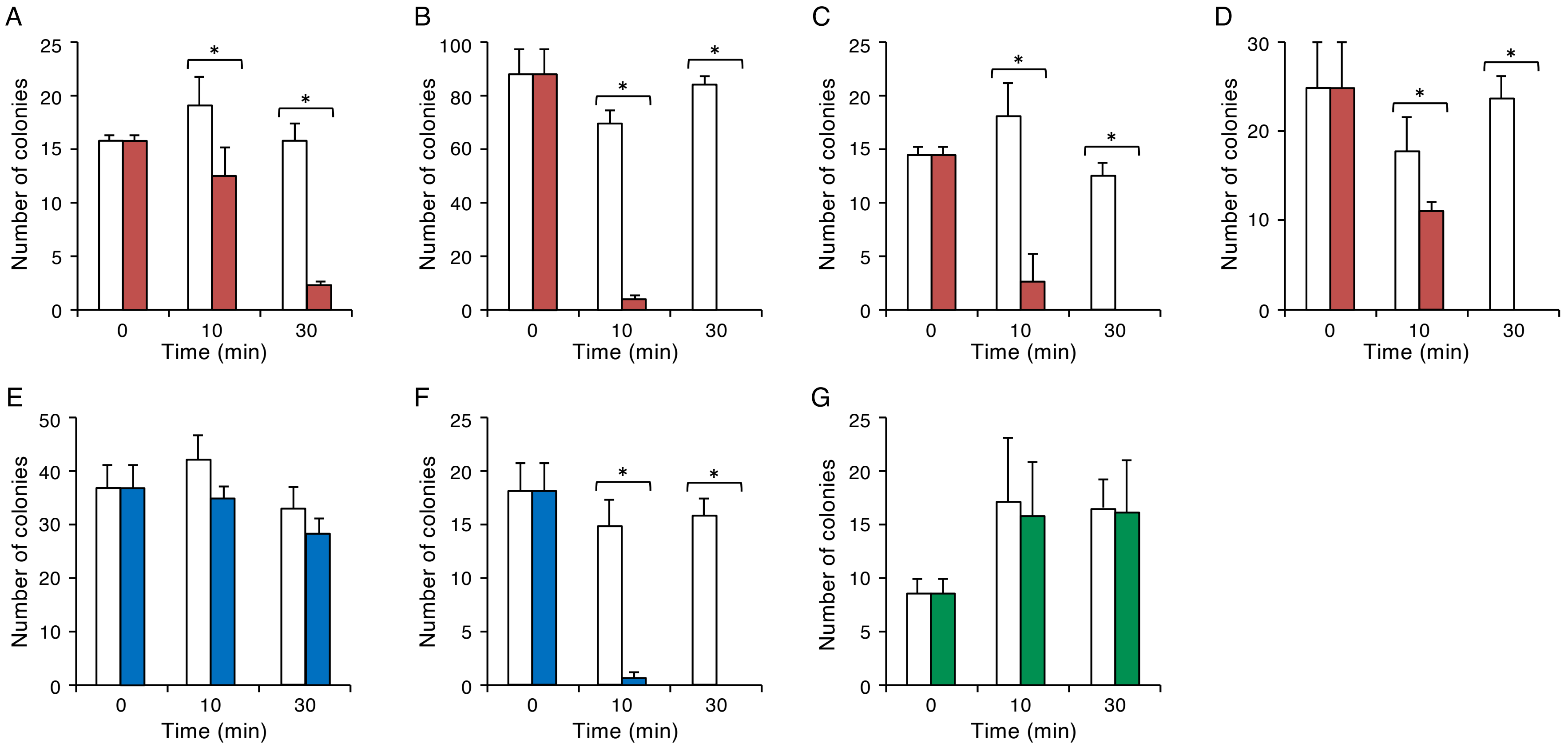

Figure S3. Killing kinetics of histone H3 against bacterial cell and fungal strains. Aliquots of gram-negative bacteria (red columns) *E. aerogenes* (A), *S. enterica* (B), *P. aeruginosa* (C), *E. coli* (D), gram-positive bacteria (blue columns) *B. cereus* (E), *S. aureus* (F) or fungus (green columns) *C. albicans* (G) were incubated at 37 °C with (colored columns) or without (blank columns) histone H3 (64 μg/mL). After 10- and 30-min incubations, suspensions were diluted and plated on trypticase soy agar plates. Viable bacterial or fungal colonies were counted after overnight growth at 37 °C. Each column and vertical bar represent the means and standard error of the mean, respectively (n = 3). Asterisks (*) indicate significant difference ($p < 0.05$) between 0 μg/mL and 64 μg/mL histone H3 treatments at each incubation time.
