# Supplementary material for: Dual Roles of Extracellular Histone H3 in Host Defense: Its Differential Regions Responsible for Antimicrobial and Cytotoxic Properties and Their Modes of Action
Source: Antibiotics (Basel). 2022 Sep 13;11(9):1240. doi: 10.3390/antibiotics11091240 (PMC9495139; doi:10.3390/antibiotics11091240)
Supplement: Supplementary file 1 [file YT.Figure.S1 Prf.pdf › YT.Figure.S1 Prf.pdf]

H3DP1 (MW 3451.94, pI 12.99, Net charge +10)

Sequence: ARTKQTARKSTGGKAPRKQLATKAARKSAPATGG

Secondary structure: CBBBBBBBCCCCCAAAAAAAAAAAAAAAAACCCCC

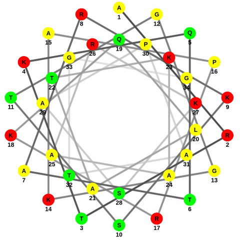

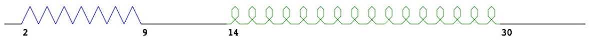

hydrophobic: 15 (44,1%)
neutral: 9 (26.5%)
hydrophilic: 10 (29,4%)

H3DP2 (MW 4178.93, pI 11.89, Net charge +8)

Sequence: VKKPHRYRPGTVALREIRRYQKSTELLIRKLPFQ

Secondary structure: AAAAAACCCCBBBBBCBBBAAAAAAAAAAAAAAA

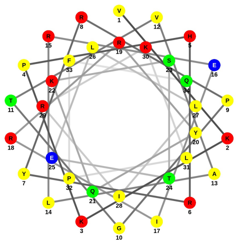

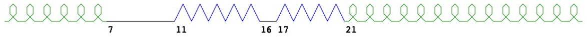

hydrophobic :14 (41.2%)
neutral: 5 (14.7%)
hydrophilic: 15 (44.1%)

H3DP3 (MW 3859.41, pI 4.77, Net charge -1)

Sequence: RLVREIAQDFKTDLRFQSSAVMALQEACEAYLVG

Secondary structure: AAAAAAAAAAABBBBBBBAAAAAAAAAAAAAAAC

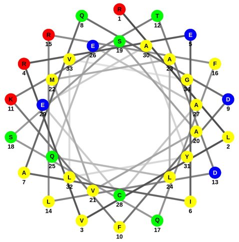

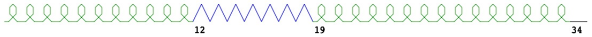

hydrophobic: 18 (52.9%)
neutral: 5 (14.7%)
hydrophilic: 11 (32.4%)

H3DP4 (MW 3836.52, pI 10.56, Net charge +4)

Sequence: LFEDTNLCAIHAKRVTIMPKDIQLARRIRGERA

Secondary structure: CCCCBBBBBBBCCBBBBBBAAAAAAAAACCCCC

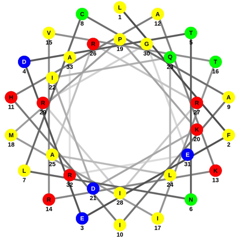

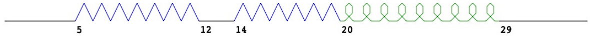

hydrophobic: 16 (48.5%)
neutral: 5 (15.1%)
hydrophilic: 12 (36.4%)

Figure S1. Amino acid sequences and compositions, molecular weight, isoelectric points, net charges, and predicted secondary structures and helical wheel projections of the histone H3-derived peptides, H3DP1–4. In the secondary structure, A, B, and C indicate α-helix, β-turn, and random coil, respectively. In the helical wheel projections, polar/basic, polar/acidic, polar/uncharged, and nonpolar amino acids are marked in red, blue, green, and yellow, respectively.
